# The Association between 25-Hydroxyvitamin D Concentration and Disability Trajectories in Very Old Adults: The Newcastle 85+ Study

**DOI:** 10.3390/nu12092742

**Published:** 2020-09-09

**Authors:** Sarah Hakeem, Nuno Mendonca, Terry Aspray, Andrew Kingston, Carmen Ruiz-Martin, Carol Jagger, John C. Mathers, Rachel Duncan, Tom R. Hill

**Affiliations:** 1 Population Health Sciences Institute, Faculty of Medical Sciences, Newcastle University, Newcastle upon Tyne NE2 4HH, UK; S.H.M.Hakeem2@ncl.ac.uk (S.H.); nuno.mendonca@nms.unl.pt (N.M.); andrew.kingston@ncl.ac.uk (A.K.); carol.jagger@ncl.ac.uk (C.J.); john.mathers@ncl.ac.uk (J.C.M.); 2 Human Nutrition Research Centre, Faculty of Medical Sciences, Newcastle University, Newcastle upon Tyne NE2 4HH, UK; 3 College of Nursing, Umm Al-Quraa University, Makkah 715, Saudi Arabia; 4 EpiDoC Unit, NOVA Medical School, Universidade Nova de Lisboa (NMS-UNL), 1150-082 Lisbon, Portugal; 5 Comprehensive Health Research Centre (CHRC), NOVA Medical School, Universidade Nova de Lisboa, 1150-082 Lisbon, Portugal; 6 Translational and Clinical Research Institute, Faculty of Medical Sciences, Newcastle University, Newcastle upon Tyne NE2 4HH, UK; Terry.Aspray@ncl.ac.uk; 7 Freeman Hospital, NHS, Newcastle upon Tyne NE7 7DN, UK; rachel.duncan2@nhs.net; 8 Bioscreening Core Facility, Faculty of Medical Sciences, Newcastle University, Newcastle upon Tyne NE4 5 PL, UK; carmen.martin-ruiz@ncl.ac.uk

**Keywords:** vitamin D status, disability, very old adults

## Abstract

*Background*: Low vitamin D status is common in very old adults which may have adverse consequences for muscle function, a major predictor of disability. *Aims*: To explore the association between 25-hydroxyvitamin D [25(OH)D] concentrations and disability trajectories in very old adults and to determine whether there is an ‘adequate’ 25(OH)D concentration which might protect against a faster disability trajectory. *Methodology*: A total of 775 participants from the Newcastle 85+ Study for who 25(OH)D concentration at baseline was available. Serum 25(OH)D concentrations of <25 nmol/L, 25–50 nmol/L and >50 nmol/L were used as cut-offs to define low, moderate and high vitamin D status, respectively. Disability was defined as difficulty in performing 17 activities of daily living, at baseline, after 18, 36 and 60 months. *Results*: A three-trajectory model was derived (low-to-mild, mild-to-moderate and moderate-to-severe). In partially adjusted models, participants with 25(OH)D concentrations <25 nmol/L were more likely to have moderate and severe disability trajectories, even after adjusting for sex, living in an institution, season, cognitive status, BMI and vitamin D supplement use. However, this association disappeared after further adjustment for physical activity. *Conclusions*: Vitamin D status does not appear to influence the trajectories of disability in very old adults.

## 1. Introduction

Life expectancy is increasing worldwide. By 2050, it is predicted that there will be 379 million people aged 80 and above, and almost 10% of the population of developed countries will be aged ≥ 80 (OECD, 2013). Disability is defined as experiencing difficulty in performing activities that are essential for independent living. Such activities comprise the basic activities of daily living (BADL), such as getting up and washing hands, and the instrumental activities of daily living (IADL), such as shopping for groceries and doing housework [1]. The frequency of ADL disability is higher among very old adults (those aged 80 and older) [2]. Difficulty with performing ADL is a predictor of longer hospital stays and of additional general practice (GP) visits [3]. Furthermore, disability increases the risk of mortality 2–3 fold among very old adults [4]. Generally, disability raises the amount of benefits paid for assistance programs and care facilities in developed countries; for example, it increases the cost of care by 22% in the United Kingdom alone [5].

Very old adults are more likely to have lower circulating concentrations of 25-hydroxyvitamin D [25(OH)D]. This is due to many reasons, including decreased production of vitamin D by skin, low exposure to sunlight, and low vitamin D intake as well as catabolism factors such as medication and disease [6]. Following hydroxylation of 25(OH)D in the kidney, 1,25(OH)D binds to its nuclear receptor (VDR) which is expressed in multiple tissues, including muscle. It then influences protein synthesis in the muscle, muscle calcium uptake and type 2 muscle’s fibre size and number [7]. Low concentrations of 25(OH)D are associated with poor muscle strength [8]. After controlling for potential confounders, 25(OH)D deficiency prevalence rates were 31 and 43% higher among men and women with muscle weakness than those with normal strength, respectively [9]. Two potential mechanisms have been suggested to explain the association between 25(OH)D and muscle function. First, age-related reduction of 1,25(OH)D reduces the stimulation of VDR expression by muscle. Second, the decline of VDR expression upon aging leads to impaired muscle response to 1,25(OH)D [10].

Maintaining adequate concentrations of 25(OH)D may protect against disability in terms of both musculoskeletal and cognitive function; the few studies that have assessed this association have found inverse associations between 25(OH)D concentration and risk of disability [8,11,12,13]. However, these studies have several limitations including: use of different definitions of low 25(OH)D concentration; being cross-sectional rather than longitudinal; recruited those aged 65 and over with few studies of the very old; being unrepresentative because they recruited women only [8,13], targeted at a specific ethnic group [12] or involved patients with a specific disease [11]. Consequently there is a need for longitudinal studies of associations between 25(OH)D concentration and disability trajectory and which focus on very-old adults, including those living in institutions.

We have previously reported vitamin D status in participants from the Newcastle 85+ study and found that 33% of the participants had vitamin D concentration < 30 nmol/L [14]. Therefore, this study aims to explore the association between 25(OH)D concentration and disability trajectory over five years in the Newcastle 85+ study participants. It also aims to investigate whether there is a threshold concentration of 25(OH)D above which the disability trajectory among the very old adults is slowed. In line with our previous work on vitamin D status and cognitive decline [15] and all-cause mortality [16] which showed that both lower and higher vitamin D status was associated with adverse biological outcomes, in the current analysis we hypothesized that lower and higher 25(OH)D concentrations are associated with a faster disability trajectory in very old adults.

## 2. Materials and Methods

### 2.1. Study Population and Design (The Newcastle 85+ Study)

The participants were from the Newcastle 85+ Study, which is socio-demographically representative study of the general UK population. It included both population-based and institutionalised older adults born in 1921 (age 85 years at recruitment) and living in Newcastle-upon-Tyne and North Tyneside (northeast England). All those who met these inclusion criteria were invited to participate (*n* = 1459). Only those individuals with end-stage terminal illness (*n* = 11) were excluded. The recruitment and baseline assessment took place over a 17-month period in 2006–2007. The follow-up phases took place at 18 (Phase 2), 36 (Phase 3) and 60 months (Phase 4) from baseline [17]. A health assessment, comprising questionnaires, measurements, function tests and a fasting blood sample, was carried out in the participants’ usual place of residence. In addition, general practice medical records were reviewed to extract data on diagnosed diseases and prescribed medication. Both the health assessment and data extraction were conducted by trained research nurses following a standard protocol. In the UK, patients are registered with a single general practice that acts as a gatekeeper to secondary care and receives details of all hospital admissions and outpatient attendance. The review of the general practice records included hospital correspondence to ensure that all recorded disease diagnoses were extracted, irrespective of where and when the diagnosis was made. Our analysis included all Newcastle 85+ Study participants (*n* = 775) for which data on health assessment, general practice records and serum 25(OH)D concentration were available at baseline (Supplemental Figure S1). From these initial group, data on health assessment and general practice records were available for 631, 484 and 344 participants at phases 2, 3, and 4, respectively.

### 2.2. Ethical Approval

The research complied with the requirements of the Declaration of Helsinki. Ethical approval was obtained from the Newcastle and North Tyneside 1 Research Ethics Committee (reference number 06/Q0905/2). Written informed consent was obtained from the participants. Where individuals lacked the capacity to give consent, for example because of cognitive impairment, a formal written opinion was sought from a relative or carer. Participants could decline to take part in any element of the study protocol.

### 2.3. Measurement of Serum 25(OH)D Concentration

Serum 25(OH)D concentration was measured at baseline from blood samples collected between June 2006 and August 2007. After an overnight fast, 40 mL blood was drawn from the antecubital vein between 7:00 and 10:30 a.m. Ninety-five per-cent of the samples were received for processing within 1 h of venepuncture. The total 25(OH)D concentration was determined by the DiaSorin radioimmunoassay (RIA) kit (DiaSorin Corporation, Stillwater, MN, USA) according to the manufacturer’s recommendations, using 25(OH)D-specific antibodies and 125I-labelled 25(OH)D (DiaSorin Corporation) as a tracer. The minimum detectable concentration of 25(OH)D was 5 nmol/L, and the inter-assay coefficients of variation ranged from 8.4% to 12.6% [18].

### 2.4. Disability Measures and Scores

At baseline and at each follow-up assessment, participants were asked about their ability to perform 17 activities comprising the basic and instrumental activities of daily living (BADLs and IADLs) and mobility items (Supplemental Table S1); these activities were taken predominantly from the Groningen Activity Restriction Scale [19]. The ability to perform the ADLs was self-reported by the participants. Each question was framed as ‘can you’ rather than ‘do you’, to assess the participants’ maximum capability to perform the activities, accounting for situational responses. Each item reported as performed without difficulty scored 0 and each item performed with difficulty scored 1 (for a maximum score of 17). A disability score was calculated based on the total number of ADLs performed with difficulty or requiring an aid/appliance or personal help [17]. Participants were classified as having a disability if they had difficulty with at least one item.

### 2.5. Other Measures/Confounders

In addition, the following variables, which are known to influence 25(OH)D concentration, were taken into account in the analysis: demographic factors [sex, living arrangements, housing type, years of full-time education], anthropometry [weight, body composition, fat-free mass, BMI] health and morbidity [disease count, cognitive status via SMMSE] and lifestyle factors [smoking, alcohol consumption, physical activity]; details on these have previously been published [20] as well as information on use of supplements containing vitamin D (yes/no) obtained from the interviewer-administrated questionnaire and prescribed vitamin D medication from the GP records [14]. However, apart from the vitamin D-containing supplements, no other supplements (including calcium, magnesium or B-vitamins) were included in the analysis owing to the very modest differences to micronutrient intakes when including supplements [21], and the inherent limitations in supplement frequency data. The date on which the blood sample was drawn was recorded and used to derive the season of collection defined as spring (March–May), summer (June–August), autumn (September–November) and winter (December–February).

### 2.6. Statistical Analysis

Normality was assessed using the Shapiro-Wilk test and confirmed using Q-Q plots and histograms. Summary statistics of normally distributed continuous values are presented as means and standard deviations (SD), and non-Gaussian distributed variables as medians and interquartile ranges (IQR). Categorical data are presented as percentages (with corresponding sample sizes).

Group-based trajectory models (GBTM) were used to derive distinct clusters of participants’ disability trajectories from baseline over the subsequent 60 months. Bayesian information criteria (BIC) were used to assess the best number of trajectories within the model. The model was then further assessed by the posterior probability of group membership >75%. Differences between disability trajectory groups were tested using the Kruskal-Wallis test for ordered non-normally distributed continuous variables (weight, BMI, fat-free mass, serum 25(OH)D, chronic disease count) and χ2 test for categorical variables (sex, physical activity, alcohol drinker, smoker, 25(OH)D, impaired cognitive status, living in an institution).

Multinomial regression was used to determine the association between disability and 25(OH)D concentration in both cross-sectional and longitudinal analysis. The concentration of 25(OH)D was not normally distributed, therefore, non-parametric analysis was used. The following cut-offs were used in the analysis: <25 nmol/L (low), 25 to 50 nmol/L (moderate) and >50 nmol/L (high) [22]. Important confounders were selected based on their clinical and theoretical relevance as well as univariate analysis with the disability trajectory. These confounders were then fitted, removed and refitted until the best possible but parsimonious model was achieved while checking for model fit statistics throughout, using 10% of change-in-estimate. The multi-collinearity between the confounders was assessed using VIF. Model 1 was an unadjusted model. Model 2 was adjusted for sex, living in an institution and season of blood collection. Model 3 was adjusted further for cognitive status, BMI and vitamin D containing medication. Model 4 was adjusted further for physical activity. The models were stratified by sex. Statistical significance was set at *p* < 0.05. All analyses were performed using IBM SPSS Statistics software version 24 (IBM, Armonk, NY, USA) except for the disability trajectory that was derived using STATA v15.0 (package traj).

### 2.7. Sensitivity Analysis

To investigate the effects of grip strength, FFM (fat-free mass) and disease count, the models were further adjusted for each of these variables. Models were rerun, excluding those participants with evidence of cognitive impairment (SMMSE score < 26). The models were also stratified by season of blood collection using the same categories as [14].

## 3. Results

### 3.1. 25(OH)D Concentration and Disability at Baseline

A cross-sectional analysis of the association between 25(OH)D cut-offs and disability baseline data reveals a U-shaped association between 25(OH)D and disability. A significant association was found between 25(OH)D concentration and disability score at baseline, (*p* = < 0.001) and (*p* = 0.002) for low (<25 nmol/L) and high (>50 nmol/L) concentrations, respectively.

### 3.2. Disability Trajectories

**Figure 1:**
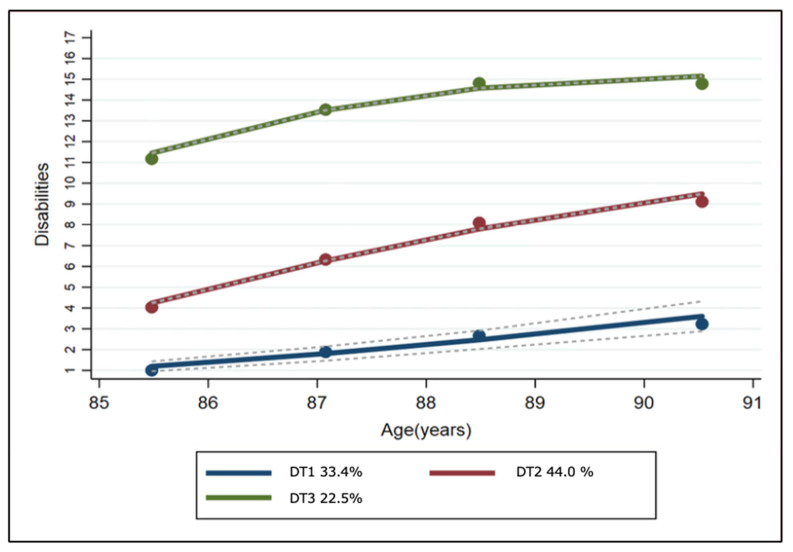
Disability trajectories with 95% confidence intervals in participants who had a serum 25(OH)D measurement available. DT1: Low to mild disability trajectory; DT2: Mild to moderate disability trajectory; DT3: Moderate to severe disability trajectory. Percentages represent group size. Disabilities resulted from calculating ADLs, IADLs and mobility. The grey dotted lines represent the 95% confidence intervals of the disability trajectories. ADL: activities of daily living, IADL: instrumental activities of daily living.

The disability trajectories (DT; one linear (low to mild trajectory) and two quadratic (mild to moderate and moderate to severe trajectories)) from age 85 to 90 years were best presented by a triple-group model. The trajectories are plotted in Figure 1 and the characteristics of the participants with each of these trajectories are described in Table 1. DT1 represents a low-to-mild disability trajectory (group size: *n* = 249–33.4%), DT2 represents a mild-to-moderate disability trajectory (group size: *n* = 351–44%) and DT3 represents a moderate-to-severe disability trajectory (group size: *n* = 175–22.5%). The participants with a low-to-mild disability trajectory had a slightly increased disability trajectory over five years, while the participants with a mild-to-moderate or moderate-to-severe disability trajectory showed serious trajectories with advancing age, with the score (number of activities that the participants were unable to undertake, unaided) increasing from four to 9.5 and from 11 to 15, respectively.

### 3.3. The Differences in Socioeconomic, Lifestyle and Health Factors between Disability Trajectories

Body weight, total number of years in education, fat-free mass and smoking did not differ significantly between participants in each of the three disability trajectories in. The participants in the three groups showed significant differences regarding their BMI, physical activity level, alcohol intake, vitamin D containing medication use, number of chronic diseases, cognitive status and living in an institution. However, the moderate-to-severe DT group was characterised by a higher percentage of women, a lower proportion of alcohol drinkers, living in an institution, being less physically active, having a higher number of chronic diseases and being cognitively impaired (Table 1). Although there were no significant differences in median serum 25(OH)D concentration between DT groups, the distribution of participants across the three categories of vitamin D adequacy based on 25(OH)D concentration (low, moderate and high) differed significantly across these three trajectories (Table 1). 

### 3.4. 25(OH)D Concentration and Disability Trajectory

The results of the analysis show that participants with low concentrations of 25(OH)D (<25 nmol/L) were more likely to have a mild-to-moderate disability trajectory (OR = 2.01, 95% CI = 1.29–3.14, *p* = 0.002) or a moderate-to-severe disability trajectory (OR = 3.39, 95% CI = 1.99–5.76, *p* = 0.001) than a low-to-mild disability trajectory compared to those with higher 25(OH)D concentrations in the unadjusted model, after adjusting for sex, living in an institution and season (OR = 2.01, 95% CI = 1.27–3.19, *p* = 0.003) and (OR = 3.02, 95% CI = 1.70–5.38, *p* = 0.001) and after further adjustment for cognitive status, BMI and vitamin D containing medication (OR = 1.97, 95% CI = 1.22–3.17, *p* = 0.005) and (OR = 3.12, 95% CI = 1.67–5.85, *p* = 0.001), respectively. However, this association disappeared after adjustment for physical activity (Table 2). The results also show that participants with high 25(OH)D concentrations were more likely to have a moderate-to-severe disability trajectory compared to those with moderate concentrations over five years but only in the unadjusted model (OR = 1.94, 95% CI = 1.23–3.06, *p* = 0.004). However, in the adjusted models, no association was found between high concentration of 25(OH)D and disability trajectory.

### 3.5. 25(OH)D Concentration and Disability Trajectory by Sex

Men with low concentrations of 25(OH)D were more likely to have a moderate-to-severe disability trajectory (OR = 3.55, 95% CI = 1.56–8.09, *p* = 0.003) than a low-to-mild disability trajectory compared to those with moderate concentrations in the unadjusted model, even after adjusting for living in an institution and season (OR = 4.42, 95% CI = 1.79–10.90, *p* = 0.001) and after further adjustment for cognitive status, BMI and vitamin D containing medication (OR = 3.83, 95% CI = 1.44–10.17, *p* = 0.007). However, this association disappeared after future adjustment for physical activity (Supplemental Table S2).

Women with low concentrations were more likely to have mild-to-moderate and moderate-to-severe disability trajectories than a low-to-mild disability trajectory compared to those with moderate concentrations in the unadjusted model (OR = 1.87, 95% CI = 1.03–3.39, *p* = 0.039) and (OR = 3.03, 95% CI = 1.50–6.13, *p* = 0.002), respectively. This association was maintained even after adjusting for sex, living in an institution and season (OR = 2.06, 95% CI = 1.12–3.83, *p* = 0.020) and (OR = 2.58, 95% CI = 1.21–5.50, *p* = 0.014), respectively. It also continued after further adjustment for cognitive status, BMI and vitamin D containing medication (OR = 1.95, 95%CI = 1.02–3.72, *p* = 0.041) and (OR = 2.70, 95% CI = 1.16–6.27, *p* = 0.020), respectively, but it disappeared after future adjustment for physical activity. Women showed a U-shaped association between 25(OH)D and a moderate-to-severe disability trajectory but only in the unadjusted model (OR = 2.29, 95% CI = 1.25–4.17, *p* = 0.007).

### 3.6. Sensitivity Analysis

Using the same models with further adjustment for grip strength, fat-free mass and disease count separately, no association was found between 25(OH)D concentration and disability trajectory. However, when physical activity was removed from the model, after adjusting for grip strength, fat-free mass and disease count, participants with a low concentration were more likely to have mild-to-moderate and moderate-to-severe disability trajectories.

The models were also rerun excluding individuals with cognitive impairment (SMMSE < 26). Participants with normal cognitive status (*n* = 561) who had a low concentration were more likely to have a moderate-to-severe disability trajectory (OR = 2.30, 95% CI = 1.15–4.58, *p* = 0.017) than a low-to-mild disability trajectory in the unadjusted model. This was also maintained in the adjusted models for the same confounders: sex, living in an institution and season (OR = 2.14, 95% CI = 1.04–4.39, *p* = 0.038) and even after adjustment for BMI and vitamin D containing medication (OR = 2.44, 95% CI = 1.13–5.27, *p* = 0.022) (Supplemental Table S3). This association disappeared after further adjustment for physical activity.

Participants’ characteristics by season have been described previously. Stratifying the analysis by season, no association was found between 25(OH)D and disability trajectory in Spring (*n* = 121). Participants with low concentrations were more likely to have a moderate-to-severe disability trajectory compared to those with moderate concentrations in the unadjusted model for Summer (*n* = 309) and Autumn (*n* = 180) (OR = 2.96, 95% CI = 1.17–7.48, *p* = 0.021) and (OR = 6.03, 95% CI = 1.47–24.77, *p* = 0.013), respectively. However, the association disappeared in the adjusted models. For Winter (*n* = 168), participants with low concentrations were more likely to have a moderate-to-severe disability trajectory (OR = 5.83, 95% CI = 1.81–18.74, *p* = 0.003) compared to normal concentrations in the unadjusted model and after adjustment for sex and living in an institution (OR = 6.44, 95% CI = 1.79–23.12, *p* = 0.004) and after further adjustment for cognitive status, BMI and vitamin D containing medication (OR = 5.10, 95% CI = 1.28–20.37, *p* = 0.021). This association disappeared after adjustment for physical activity. On the other hand, participants with high concentrations were more likely to have a moderate-to-severe disability trajectory (OR = 6.92, 95% CI = 2.23–21.43, *p* = 0.001) compared to normal concentrations in the unadjusted model and after adjustment for sex, living in an institution and season (OR = 4.51, 95% CI = 1.24–16.37, *p* = 0.022). After adjustment for cognitive status, BMI and vitamin D containing medication, the association was attenuated although it became stronger after adjustment for physical activity (OR = 6.11, 95% CI = 1.01–36.75, *p* = 0.048) (Supplemental Table S4).

Serum 25(OH)D was used as a continuous variable in the analysis, but no association was found between 25(OH)D and disability (baseline data) or disability trajectory (follow up phase data) in the unadjusted or adjusted models.

## 4. Discussion

### 4.1. Main Findings

For the current analysis, the disability trajectories model was best presented by the triple-group model. These trajectories differed from two previously derived disability trajectories in different samples of the Newcastle 85+ Study [23,24]. We showed that, in partially adjusted models, people aged 85+ years with a 25(OH)D concentration (25–50 nmol/L) were more likely to have less disability at baseline and a slower disability trajectory over the following five years. However, in fully adjusted models, our results did not show a protective effect of any 25(OH)D concentration on trajectories of disability over five years.

### 4.2. Evidence from Other Studies

The lack of an association between 25(OH)D concentration and disability trajectories in our study is inconsistent with the findings of prospective cohort studies that investigate the association between vitamin D status and disability in those age 65 years and over. For example, two studies found that a 25(OH)D < 50 nmol/L increased the risk of disability in arthritis and multiple sclerosis patients, respectively [11,12]. Likewise, Semba, et al. [13] found that a 25(OH)D < 50 nmol/L was associated with a higher possibility of disability in women aged over 65 years and living in the community. The higher risk of a disability trajectory amongst participants with a concentration higher than 50 nmol/L in the cross-sectional analysis and in unadjusted models in the prospective analyses could be driven largely by those with cognitive impairment or those taking vitamin D containing medication or prescribed medication. First, our overall analysis showed that the association between a high concentration and disability trajectory disappeared after adjusting for these variables. Moreover, excluding participants with low SMMSE scores supports this finding. On the other hand, participants with a normal cognitive status did not show an association between high concentration and disability trajectories. In addition, there is no general agreement amongst researchers regarding the optimal concentration of 25(OH)D in relation to disability trajectory. The Institute of Medicine (IoM) defines vitamin D deficiency as a concentration of 25(OH)D < 30 nmol/L, and vitamin D adequacy as a concentration of >50 nmol/L for all age groups based on integrating data from several health outcomes and PTH [25]. In contrast, the UK Scientific Advisory Committee on Nutrition (SACN) defines vitamin D deficiency at a 25(OH)D concentration < 25 nmol/L [26]. However, our previous findings from the Newcastle 85+ study have documented a U-shaped association between 25(OH)D and muscle strength and performance [27].

Generally, the main role of vitamin D is to support musculoskeletal health. Therefore, maintaining an moderate 25(OH)D concentration is essential in order to slow the effect of ageing on the bones and muscles. Ageing is accompanied by a redistribution of the cortical and trabecular bone [28]. Moreover, a low 25(OH)D concentration increases osteoblastic activity and bone turnover [29,30]. A significant positive association has also been documented between 25(OH)D concentration, BMD [31] and type II muscle fibre [32] in older people. In addition, the VDR expression is reduced in the muscles as part of ageing [33]. A positive association between 25(OH)D concentration and muscle strength has been reported [32]. Therefore, a lack of VDR, which is expected in very-old adults, leads to reduced muscle mass and strength, as explained previously. Furthermore, studies in rats have demonstrated that a high PTH, due to a low concentration of 25(OH)D, induces muscle catabolism and reduces calcium transport in the skeletal muscle [32], thereby leading to low muscle strength. Combined, this can explain the effect of a low concentration of 25(OH)D on the onset and progression of disability.

In addition, the association between moderate 25(OH)D concentration and physical performance and strength has been confirmed previously [10]. Kotlarczyk, et al. [34] found that slower gait speed and lower IADL scores were associated with low 25(OH)D concentration. Moreover, a positive association between the 8-foot walk test and the sit-to-stand test, with a concentration of 25(OH)D, was also found [35]. These results indicate that a low concentration of 25(OH)D was associated with low muscle strength, which is a predictor of disability. Consistent with our results, Granic, et al. [27] demonstrated that a 25(OH)D concentration of > 30 nmol/L maintains muscle strength, but a concentration of > 50 nmol/L did not have further muscular or musculoskeletal benefits in the very old adults. However, whilst we appreciate that muscle function and disability per se are different parameters, a comparison nonetheless is relevant because of the role of muscle function in contributing to disability.

Physical activity is clearly a predictor of disability [11], although the association between PA and 25(OH)D was conflicted between the studies. First, a high concentration of 25(OH)D can positively influence the intensity of PA [36]. However, a converse association is also suggested [37]. In the same vein, a study analysing the data from NHANES reported that PA is generally associated with a high concentration of 25(OH)D, whether this activity occurs indoors or outdoors [38]. Therefore, restricted PA, which is associated with disability, can have an adverse effect on 25(OH)D concentration, possibly due either to a defect in metabolism or limited exposure to sunlight. Besides, PA is accompanied by improved health, stronger muscles and a lower BMI, which are all associated with 25(OH)D concentration [13,39,40]. Furthermore, the progression of disability is accompanied by a greater risk of feeding disability onset [41]; this contributes to the risk of nutrient deficiency, including vitamin D. Our results show that the association between 25(OH)D and the disability trajectory disappeared after adjusting for PA. This suggests that the association between 25(OH)D concentration and disability could be due the effect of PA rather than 25(OH)D concentration. This means those with a higher PA have a better vitamin D status and, obviously, those with a high PA have less disability.

Age-related changes also result in body composition changes. For that reason, a lean body mass is significantly lower in older adults compared to younger ones—a change that accelerates after the age of 60 [42]. The univariate analysis of our data showed an association between fat-free mass and disability trajectories. However, the evidence demonstrated that the amount of fat mass but not fat-free mass was associated with muscle function and disability. For instance, Sternfeld, et al. [43] and Visser, et al. [44] agreed that there was no association between physical disability and total body skeletal muscle mass, while a high percentage of fat mass was associated with physical disability.

Even though a higher lean-to-fat ratio was associated with a faster walking speed, this suggests that the impact of a lean mass is important in relation to the amount of body fat. Indeed, fat-free mass was not a significant predictor of mobility-related disability in the regression model [45]. The association between 25(OH)D concentration and fat and lean mass could be explained by the escalation in fat mass, which may enhance the storage of vitamin D and, consequently, lower the circulating 25(OH)D [40]. However, the adjustment for BMI in the model, and for FFM in the sensitivity analysis, did not affect the association between 25(OH)D concentration and disability trajectory in the current study.

Our results also suggest that in partially adjusted models, men with a low concentration of 25(OH)D were more likely to develop only a severe disability trajectory, while women with a low concentration were more likely to develop either a moderate or a severe disability trajectory. This could be explained by the findings of Granic, et al. [46], who demonstrated that men had better muscle strength and physical performance (measured by grip strength and timed-up-and-go), but a steeper decline in both grip strength and timed-up-to-go over five years. Similarly, Millán-Calenti et al. [3]. reported that older men and women (80+ years) have a higher risk of being dependent (OR = 1.10) using ADL and IADL compared to younger adults (65+), but the risk among women is even higher (OR = 2.48). Conversely, our results are inconsistent with the findings of Semba, et al. [13], who demonstrated that only women with a low 25(OH)D concentration were at risk of having a disability. This difference could be due to the smaller number of men included in the studies compared to women.

The association between 25(OH)D concentration and disability trajectories in the current study varies by season. In the spring, no association was found, whereas a significant adverse association was found between a 25(OH)D concentration lower than 25 nmol/L and disability trajectory, but only in the unadjusted model. However, in Winter, a U-shaped association was found between 25(OH)D concentration and disability trajectory. This conflicting results between the seasons could be explained by the differences between the participants’ cognitive status, PA and vitamin D containing medication usage. The total number of participants in the Spring was the lowest (*n* = 121); consequently, Spring was associated with the lowest percentage of participants who had normal cognitive status (14.5%), were physically active (14.5%) and who took vitamin D containing medication (15.5%) when compared to the other seasons. This may explain the failure to detect the association. On the contrary, the data showed that, in the Winter, of the 165 participants, 14 were cognitively impaired and took vitamin D containing medication; nine of the cognitively impaired participants were physically active compared to the 52 participants who had a normal cognitive status and were physically active. Therefore, a potential negative effect of the highest 25(OH)D tertile on disability trajectory could be partly driven by those who have an impaired cognitive status, that influences their PA, and by those who have reached a higher concentration through taking vitamin D containing medication shortly before the baseline assessments.

### 4.3. Strengths and Limitations

Our study has several strengths, including its prospective design, its broad representativeness of the population in England and Wales, large number of participants, the five-year follow up to measure disability, the robustness of the clustering technique (GBTM) used to derive disability trajectory, and the adjustment for several potential confounders associated with disability and 25(OH)D concentration. Physical activity and season, which could reflect UV exposure, were also considered in the models. Determining the disability by using 17 ADLs that compromise BADL, IADL and mobility items is also a strength of this study. Moreover, our study used prevalent cut-offs of serum 25(OH)D to determine the concentration required to predict the onset and progress of disability trajectory.

However, the findings reported here should be interpreted with caution due to the following limitations. First, the concentration of 25(OH)D was only measured at baseline, so it might change during the subsequent five years depending on sun exposure, season, supplement intake, physical activity and disease. However, the changes in these variables are unlikely across the follow-up phases, if only disease/disability may increase but not the others. Another limitation was that the frequency or dose of supplements used as well as UV exposure were not measured. Finally, it is possible that some disability transitions were not fully captured during the follow up phases, as these were 18 or 24 months apart.

## 5. Conclusions

We found a U-shaped association between 25(OH)D and disability at baseline with both low (<25 nmol/L) and high (>50 nmol/L) 25(OH)D concentrations associated with faster disability. However these findings should be interpreted with caution as residual confounding is very likely driving these associations. In fully adjusted models, we failed to find a significant association between any 25(OH)D concentration and disability trajectories over 5 years. However, it should be noted that sample size limitations may have precluded the detection of statistically significant findings and that larger studies are needed for this research question.

## Figures and Tables

**Table 1:** Participant characteristics by the three disability trajectories identified at baseline.

	Low-to-Mild (*n* = 249)	Mild-to-Moderate (*n* = 351)	Moderate-to-Severe (*n* = 175)	*p*
Women % (*n*)	48.4 (121)	56.6 (231)	69.3 (122)	<0.001
Weight (kg) mean (SD)	63.9 (11.8)	63.5 (13.4)	63.9 (14.3)	0.732
BMI mean (SD)	23.8 (3.8)	24.7 (4.4)	24.9 (5.2)	0.029
Fat-free mass (kg) mean (SD)	46.5 (9.2)	44.4 (9.1)	45 (8.9)	0.151
Total number of years in education % (*n*) 0–9 years 10–11 years 12–20 years	61.9 (153) 23.9 (59) 14.2 (35)	62.1 (213) 24.2 (83) 13.7 (47)	70.3 (111) 22.2 (35) 7.6 (12)	0.241
Physical activity % (*n*) Low Moderate High	2.4 (6) 15.8 (55) 63.2 (110)	27.3 (68) 58.7 (205) 33.9 (59)	70.3 (175) 25.5 (89) 2.9 (5)	<0.001
Alcohol drinkers % (*n*)	80 (156)	72.4 (168)	55.3 (52)	<0.001
Smoking % (*n*)	3.6 (9)	8 (28)	4.5 (8)	0.124
Vitamin D containing medication % (*n*)	10 (25)	13.4 (47)	31.8 (56)	<0.001
Supplement users % (*n*)	23.3 (58)	20.8 (73)	12 (21)	0.012
Serum 25(OH)D nmol/L median (IQR)	42 (29–59)	36 (23–58)	39 (21–70)	0.178
25(OH)D <25 nmol/L (low) % (*n*) 25–50 nmol/L (moderate) % (*n*) >50 nmol/L (high) % (*n*)	26.4 (66) 34.7 (122) 38.1 (67)	36 (90) 31.8 (112) 19.9 (35)	37.6 (94) 33.5 (118) 42 (74)	0.02
Chronic disease count mean (SD)	4.1 (1.5)	4.93 (1.75)	5.6 (1.9)	<0.001
Impaired cognitive status % (*n*)	12 (30)	23.3 (82)	57.5 (100)	<0.001
Living in institution % (*n*)	0.4 (1)	3.4 (12)	30.7 (54)	<0.001

BMI: body mass index. SD: standard deviations. IQR: medians and interquartile ranges. *p*, *p*-value: Kruskal-Wallis test for continuous non-normally distributed variables or χ2 test for categorical variables 25(OH)D: <25 nmol/L (low), 25–50 nmol/L (moderate), >50 nmol/L (high).

**Table 2:** Association between 25(OH)D concentration and disability trajectories.

Trajectories	25(OH)D	Model 1			Model 2			Model 3			Model 4		
		OR	95% CI	*p*	OR	95% CI	*p*	OR	95% CI	*p*	OR	95% CI	*p*
DT1: Low-to-mild	(ref)	(ref)			(ref)			(ref)			(ref)		
DT2: Mild-to-moderate	<25 nmol/L	2.01	1.29–3.14	0.002	2.01	1.27–3.19	0.003	1.97	1.22–3.17	0.005	1.61	0.95–2.74	0.074
	25–50 nmol/L	(ref)			(ref)			(ref)			(ref)		
	>50 nmol/L	1.05	0.73–1.52	0.774	0.94	0.64–1.38	0.771	0.92	0.61–1.38	0.707	1.07	0.69–1.67	0.749
DT3: Moderate-to-severe	<25 nmol/L	3.39	1.99–5.76	0.001	3.02	1.70–5.38	0.001	3.12	1.67–5.85	0.001	1.95	0.94–4.06	0.071
	25–50 nmol/L	(ref)			(ref)			(ref)			(ref)		
	>50 nmol/L	1.94	1.23–3.06	0.004	1.34	0.80–2.22	0.254	0.83	0.45–1.55	0.577	1.02	0.49–2.12	0.945

CI: confidence interval. BMI: body mass index. OR: odd ratio. ref: reference. 25(OH)D cut-offs: <25 nmol/L (low), 25–50 nmol/L (moderate) and >50 nmol/L (high); Number of participants with low, moderate and high 25(OH)D for DT1 is 66, 122, 67; DT2 is 89, 111, 34; DT3 is 94, 118, 74; Model 1 is the unadjusted model. Model 2 is further adjusted for sex, living in an institution and season. Model 3 is further adjusted for cognitive status, BMI and vitamin D containing medication. Model 4 is further adjusted for physical activity.

## Supplementary Materials

The following are available online at https://www.mdpi.com/2072-6643/12/9/2742/s1, Supplementary Figure S1: The Newcastle 85+ Study recruitment. Supplementary Table S1. Self-reported activities of daily living. Supplementary Table S2. Association between different 25(OH)D cut-offs and disability trajectories by sex. Supplementary Table S3. Association between 25(OH)D concentration and disability trajectories of people with normal cognitive status. Supplementary Table S4. Association between different 25(OH)D cut-offs and disability trajectories by season.
