# Supplementary material for: The Association between 25-Hydroxyvitamin D Concentration and Disability Trajectories in Very Old Adults: The Newcastle 85+ Study
Source: Nutrients. 2020 Sep 9;12(9):2742. doi: 10.3390/nu12092742 (PMC7551468; doi:10.3390/nu12092742)
Supplement: Supplementary file 1 [file nutrients-12-02742-s001.pdf]

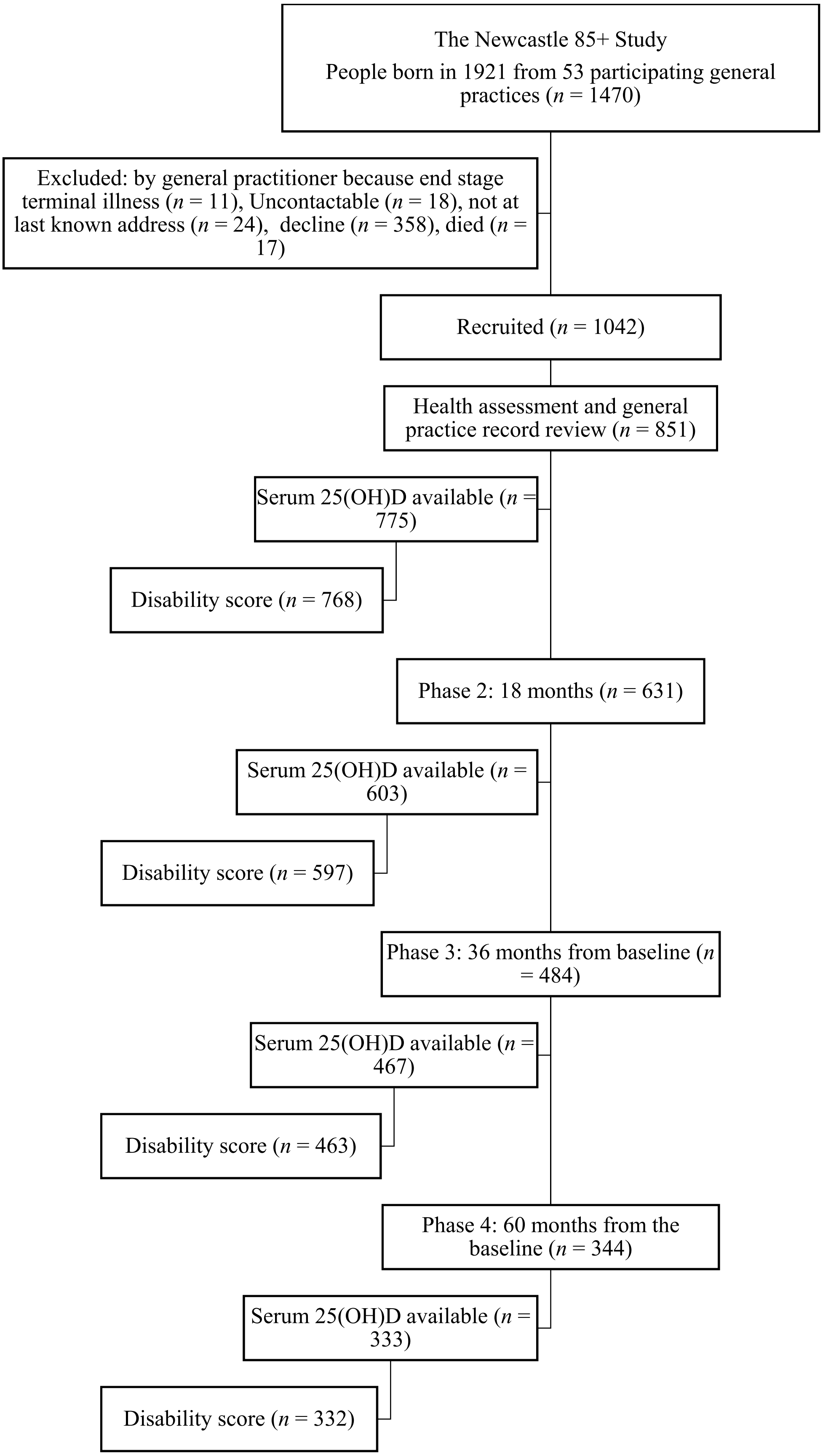

Figure S1: The Newcastle 85+ Study recruitment

Table S1. Self-reported activities of daily living

BADL	IADL	Mobility items	Response categories
- Feeding self - including cutting up of food<br>- Washing face and hands<br>- Washing all over<br>- Getting in and out of bed<br>- Getting on and off the toilet<br>- Getting in and out of a chair<br>- Dressing and undressing<br>- Cutting own toenails	- Light housework<br>- Heavy housework<br>- Preparing and cooking a hot meal<br>- Shopping for groceries<br>- Taking medication<br>- Managing money	- Getting around the house<br>- Going up and down stairs/steps<br>- Walking at least 400 yards	- Can do on own without difficulty<br>- Can do on own but with difficulty<br>- Can do on own but only with aid or appliance unable to do without personal help

*Reference [46]. BADL: basic activities of daily living. IADL: instrumental activities of daily living.

Table S2. Association between different 25(OH)D cut-offs and disability trajectories by sex

Sex	**Traj**	**25(OH)D**	**Model 1**			**Model 2**			**Model 3**			**Model 4**		
			**OR**	**95%CI**	***p***	**OR**	**95%CI**	***p***	**OR**	**95%CI**	***p***	**OR**	**95%CI**	***p***
Men (n = 304)	DT1	(ref)	(ref)			(ref)			(ref)			(ref)		
		<25nmol/l	1.96	0.99-3.89	0.052	1.94	0.97-3.89	0.060	2.01	0.98-4.09	0.054	1.58	0.71-3.49	0.255
	DT2	25-50 nmol/l	(ref)			(ref)			(ref)			(ref)		
		>50nmol/l	0.46	0.46-1.46	0.512	0.82	0.45-1.48	0.518	0.84	0.44-1.57	0.586	1.26	0.63-2.51	0.500
		<25nmol/l	3.55	1.56-8.09	0.003	4.42	1.79-10.90	0.001	3.83	1.44-10.17	0.007	1.67	0.53-5.25	0.378
	DT3	25-50 nmol/l	(ref)			(ref)			(ref)			(ref)		
		>50nmol/l	1.14	0.53-2.46	0.726	0.79	0.32-1.96	0.625	0.41	0.14-1.23	0.115	0.76	0.20-2.81	0.687
Women (n = 471)	DT1	(ref)	(ref)			(ref)			(ref)			(ref)		
		<25nmol/l	1.87	1.03-3.39	0.039	2.06	1.12-3.82	0.020	1.95	1.02-3.72	0.041	1.59	0.77-3.28	0.202
	DT2	25-50 nmol/l	(ref)			(ref)			(ref)			(ref)		
		>50nmol/l	1.13	0.68-1.86	0.622	1.10	0.66-1.82	0.699	1.01	0.58-1.77	0.946	0.96	0.52-1.77	0.896
		<25nmol/l	3.03	1.50-6.13	0.002	2.58	1.21-5.50	0.014	2.70	1.16-6.27	0.020	2.05	0.77-5.46	0.150
	DT3	25-50 nmol/l	(ref)			(ref)			(ref)			(ref)		
		>50nmol/l	2.29	1.25-4.17	0.007	1.69	0.89-3.23	0.107	1.18	0.53-2.62	0.684	1.10	0.43-2.80	0.838

DT1, low-to-mild disability trajectory; DT2, mild-to-moderate disability trajectory; DT3 moderate-to-severe disability trajectory. BMI: body mass index. ref: reference. 25(OH)D: <25 nmol/l (low), 25-50 nmol/l (moderate), >50nmol/l (high). OR, odd ratio. CI, confidence interval. *p*, *p*-value. Model 1 is the unadjusted model. Model 2 is further adjusted for living in an institution and season. Model 3 is further adjusted for cognitive status, BMI, and vitamin D containing medication. Model 4 is further adjusted for physical activity.

Table S3. Association between 25(OH)D concentration and disability trajectories of people with normal cognitive status

**Traj**	**25(OH)D**	**Model 1**			**Model 2**			**Model 3**			**Model 4**		
		**OR**	**95%CI**	*p*	**OR**	**95%CI**	*p*	**OR**	**95%CI**	*p*	**OR**	**95%CI**	*p*
DT1	(ref)	(ref)			(ref)			(ref)			(ref)		
DT2	<25nmol/l	1.45	0.90-2.35	0.124	1.49	0.90-2.47	0.114	1.40	0.83-2.35	0.203	1.11	0.62-1.99	0.718
	25-50 nmol/l	(ref)			(ref)			(ref)			(ref)		
	>50nmol/l	0.98	0.65-1.46	0.923	0.90	0.59-1.37	0.642	0.87	0.56-1.35	0.536	1.03	0.63-1.67	0.892
DT3	<25nmol/l	2.30	1.15-4.58	0.017	2.14	1.04-4.39	0.038	2.44	1.13-5.27	0.022	1.41	0.58-3.40	0.444
	25-50 nmol/l	(ref)			(ref)			(ref)			(ref)		
	>50nmol/l	1.37	0.74-2.55	0.310	1.18	0.62-2.25	0.608	0.89	0.43-1.86	0.773	1.11	0.47-2.59	0.807

DT1, low-to-mild disability trajectory; DT2, mild-to-moderate disability trajectory; DT3 moderate-to-severe disability trajectory. BMI: body mass index. ref: reference. 25(OH)D: <25 nmol/l (low), 25-50 nmol/l (moderate), >50nmol/l (high). OR, odd ratio. CI, confidence interval. *p*, *p*-value. Model 1 is the unadjusted model. Model 2 is further adjusted for sex, living in an institution and season. Model 3 is further adjusted for BMI, and vitamin D containing medication. Model 4 is further adjusted for physical activet.

Table S4. Association between different 25(OH)D cut-offs and disability trajectories by season.

season	Traj	25(OH)D	Model 1			Model 2			Model 3			Model 4		
			OR	95%CI	p	OR	95%CI	p	OR	95%CI	p	OR	95%CI	p
Spring (*n* = 121)	DT1	(ref)	(ref)			(ref)			(ref)			(ref)		
	DT2	<25nmol/l	1.77	0.68-4.55	0.236	1.73	0.66-4.49	0.258	1.71	0.63-4.65	0.290	1.76	0.54-5.72	0.342
		25-50 nmol/l	(ref)			(ref)			(ref)			(ref)		
		>50 nmol/l	0.85	0.27-2.62	0.778	0.72	0.22-2.32	0.583	0.80	0.51-4.28	0.800	1.14	0.17-7.47	0.891
	DT3	<25nmol/l	2.83	0.97-9.13	0.081	2.98	0.81-10.95	0.100	4.71	0.92-24.11	0.063	3.52	0.49-24.95	0.208
		25-50 nmol/l	(ref)			(ref)			(ref)			(ref)		

		>50 nmol/l	2.15	0.59-7.89	0.245	0.97	0.19-4.95	0.973	1.75	0.14-21.78	0.662	3.31	0.13-83.02	0.466
Summer (*n* = 309)	DT1	(ref)	(ref)			(ref)			(ref)			(ref)		
		<25nmol/l	1.97	0.92-4.25	0.080	1.80	0.83-3.92	0.136	1.86	0.83-4.15	0.128	1.26	0.50-3.15	0.618
	DT2	25-50 nmol/l	(ref)			(ref)			(ref)			(ref)		
		>50 nmol/l	0.98	0.56-1.71	0.945	0.94	0.53-1.65	0.831	0.99	0.54-1.82	0.989	1.13	0.56-2.27	0.714
		<25nmol/l	2.96	1.17-7.48	0.021	2.53	0.95-6.72	0.062	2.56	0.90-7.28	0.076	1.17	0.33-4.15	0.800
	DT3	25-50 nmol/l	(ref)			(ref)			(ref)			(ref)		
		>50 nmol/l	1.35	0.65-2.80	0.420	1.07	0.48-2.35	0.865	0.88	0.344-2.26	0.794	0.94	0.30-2.85	0.913
Autumn (*n* = 180)	DT1	(ref)	(ref)			(ref)			(ref)			(ref)		
		<25nmol/l	3.43	0.90-13.12	0.071	3.80	0.96-15.10	0.057	3.81	0.70-20.71	0.121	2.24	0.34-14.63	0.398
	DT2	25-50 nmol/l	(ref)			(ref)			(ref)			(ref)		
		>50 nmol/l	1.01	0.48-2.14	0.963	0.93	0.43-2.01	0.857	0.83	0.36-1.93	0.680	1.11	0.42-2.92	0.832
		<25nmol/l	6.03	1.47-24.77	0.013	3.09	0.66-14.40	0.149	4.26	0.65-27.80	0.129	1.38	0.14-13.46	0.778
	DT3	25-50 nmol/l	(ref)			(ref)			(ref)			(ref)		
		>50 nmol/l	1.49	0.62-3.53	0.366	0.95	0.37-2.44	0.921	0.30	0.08-1.06	0.062	0.26	0.049-1.43	0.123
Winter (*n* = 168)	DT1	(ref)	(ref)			(ref)			(ref)			(ref)		
		<25nmol/l	2.15	0.91-5.07	0.079	2.08	0.88-4.95	0.094	2.09	0.84-5.19	0.109	2.05	0.77-5.47	0.148
	DT2	25-50 nmol/l	(ref)			(ref)			(ref)			(ref)		
		>50 nmol/l	1.43	0.59-3.44	0.422	1.26	0.51-3.10	0.606	1.16	0.41-3.22	0.774	1.65	0.27-4.95	0.367
		<25nmol/l	5.83	1.81-18.74	0.003	6.44	1.79-23.12	0.004	5.10	1.28-20.37	0.021	4.38	0.96-19.97	0.056
	DT3	25-50 nmol/l	(ref)			(ref)			(ref)			(ref)		

>50 nmol/l	6.92	2.23-21.43	0.001	4.51	1.24-16.37	0.022	3.82	0.79-18.35	0.094	6.11	1.01-36.75	0.048

DT1, low-to-mild disability trajectory; DT2, mild-to-moderate disability trajectory; DT3 moderate-to-severe disability trajectory. BMI: body mass index. ref: reference. 25(OH)D: <25 nmol/l (low), 25-50 nmol/l (moderate), >50nmol/l (high). OR, odd ratio. CI, confidence interval. *p*, *p*-value. Model 1 is the unadjusted model. Model 2 is further adjusted for sex, living in an institution. Model 3 is further adjusted for cognitive status, BMI, and vitamin D containing medication. Model 4 is further adjusted for physical activity.
